# Catastrophic Mid-Flexion Instability After Avulsion Fractures of the Articular Capsule of the Femur and Tibia in a Patient With Posterior-Stabilized Total Knee Arthroplasty: A Case Report

**DOI:** 10.7759/cureus.44379

**Published:** 2023-08-30

**Authors:** Akihiro Itamoto, Kohei Nishitani, Shinichi Kuriyama, Shinichiro Nakamura, Shuichi Matsuda

**Affiliations:** 1 Department of Orthopaedic Surgery, Kyoto University Graduate School of Medicine, Kyoto, JPN

**Keywords:** joint capsule, avulsion fracture, subluxation, rotating-hinge, mid-flexion instability, complication, osteoarthritis, revision, total knee arthroplasty

## Abstract

Mid-flexion instability can be caused by patient-related, implant-related, or technique-specific factors and impairs the activities of daily living after total knee arthroplasty (TKA). In this study, we report a rare case of a patient with severe mid-flexion instability following tibial and femoral avulsion fractures after posterior-stabilized (PS) TKA for knee osteoarthritis. An 82-year-old female with bilateral knee osteoarthritis underwent staged bilateral TKA with a posterior-stabilized prosthesis. The course of the early postoperative period was good, and the patient was able to walk independently with a cane. Two months postoperatively, the patient fell and then experienced left knee pain and instability in the mid-flexion range. Radiographic images showed avulsion fractures of the articular capsule of the femur and tibia, and fluoroscopic examination showed severe posterior subluxation of the tibia between 40° and 60° of flexion. Conservative treatment with a functional knee brace and quadriceps training was initiated due to the patient’s hesitation to undergo a second surgery; however, no improvement was observed. Eventually, revision surgery was planned three months after the fall incident (five months after the left primary TKA). At revision surgery, osteosynthesis of the tibial avulsion fracture and thickening of the PS insert did not sufficiently stabilize the instability, and revision TKA with a rotating-hinge prosthesis was needed. The postoperative course was uneventful, and she was able to walk with a cane within two weeks after revision TKA with no complaints of instability. Two years postoperatively, the patient recovered well and had no recurrence of instability, pain, or dysfunction. This case report shows that loss of support by the joint capsules due to avulsion fractures may cause significant anteroposterior instability in the mid-flexion position after posterior-stabilized TKA. In such a case, conservative treatment failed, and the revision of the rotating-hinge prosthesis provided stability and good improvement.

## Introduction

Multiple registries have reported that 5% to 14% of first-revision total knee arthroplasties (TKAs) are due to instability [1-3]. Post-TKA instability, which causes just a vague sense in a mild case but causes pain, giving way, or dislocation in a severe case, is classified into five categories: extension, flexion, mid-flexion, genu recurvatum, and global instability [4]. Mid-flexion instability following TKA, where the knee is stable at extension and 90° of flexion but unstable somewhere between these 2 points, is one of the major causes of decreased patient satisfaction and revision TKA [5,6]. Trauma, such as ligament injuries and fractures, can cause post-traumatic TKA instability. In revision cases due to flexion instability in TKA for failed non-operative treatment, about 3% of patients had a history of trauma [7]. However, mid-flexion instability after trauma is rare. In this study, we report a patient with severe post-traumatic mid-flexion instability after posterior-stabilized (PS) TKA for medial compartment osteoarthritis of the knee following tibial and femoral avulsion fractures.

## Case presentation

**Figure 1:**
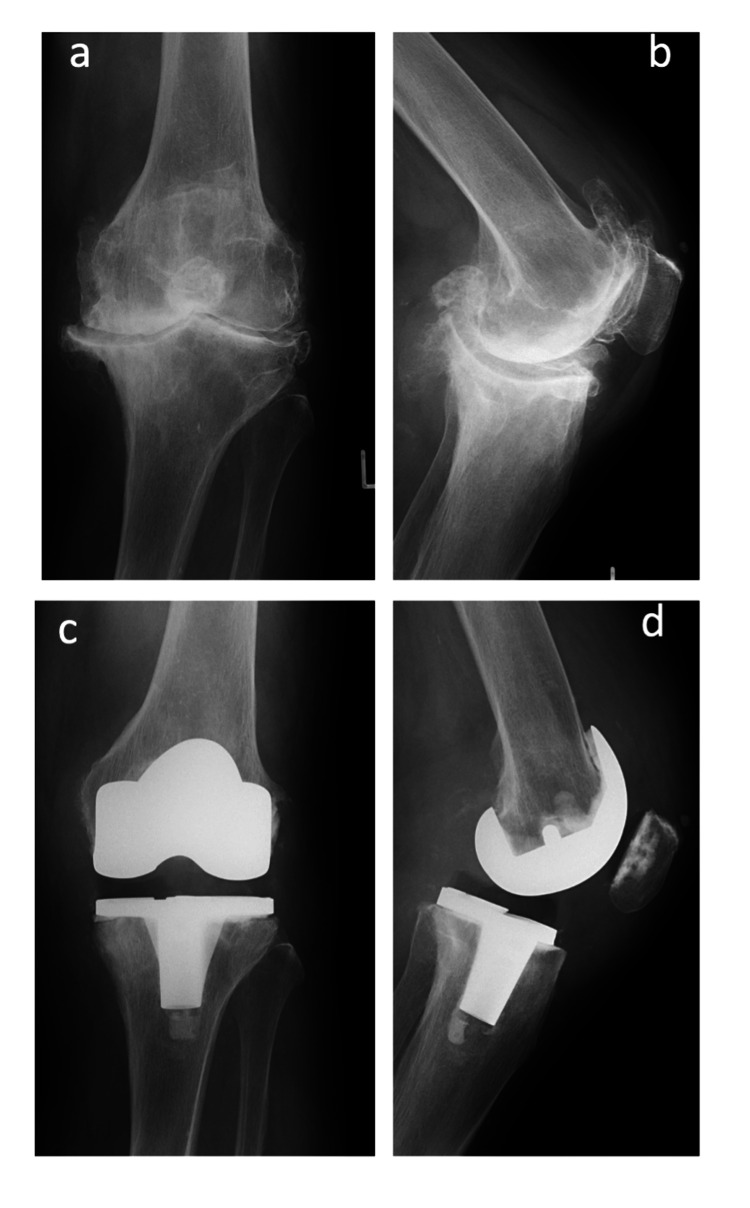
Knee radiographs before the fall. Preoperative anteroposterior (a) and lateral (b) radiographs of the left knee showed end-stage knee osteoarthritis. Anteroposterior (c) and lateral (d) radiographs after primary total knee arthroplasty.

Data collection was approved by the Kyoto University Graduate School and Faculty of Medicine Ethics Committee, and written informed consent was obtained from the patient for the publication of the case report. An 82-year-old female with hypertension had bilateral knee pain for six years before visiting our hospital. She was 152.2 cm tall and weighed 54.1 kg, with a body mass index of 23.4 kg/m2. The range of motion (ROM) of the knee joints was 10-95° on both sides. Radiographs showed bilateral knee osteoarthritis of Kellgren-Lawrence grade 4 in both knees [8], with a hip-knee angle (HKA) of 20.7° varus and 20.6° varus on the left and right sides (Figure 1a-1b). Varus and valgus stress radiographs revealed no obvious collateral ligament relaxation. The Knee Society Score-Knee Score (KSS-KS) [9] was 49 and 34 points, and the Function Score (FS) was 45 and 45 points on the left and right sides, respectively. She was diagnosed with bilateral end-stage knee osteoarthritis; thus, staged bilateral TKA was performed on the right knee first, with a four-month interval using PS implants (NexGen LPS flex, Insert: 12 mm, Zimmer-Biomet, Warsaw, IN, USA), aiming mechanical alignment using measured resection technique (Figure 1c-1d). The postoperative radiograph revealed that the HKA was 3.8° and 4.7° varus on the left and right sides, respectively. The left tibial component was implanted at a 0.8° internally rotated position to Akagi’s line, showing no excessive internal rotation. The course of the early postoperative period was good, and the patient was able to walk independently with a cane. At the time of discharge, three weeks after left TKA (approximately five months after right TKA), the KSS-KS was 79 and 93 points, and the FS was 55 and 55 points on the left and right sides, respectively.

**Figure 2:**
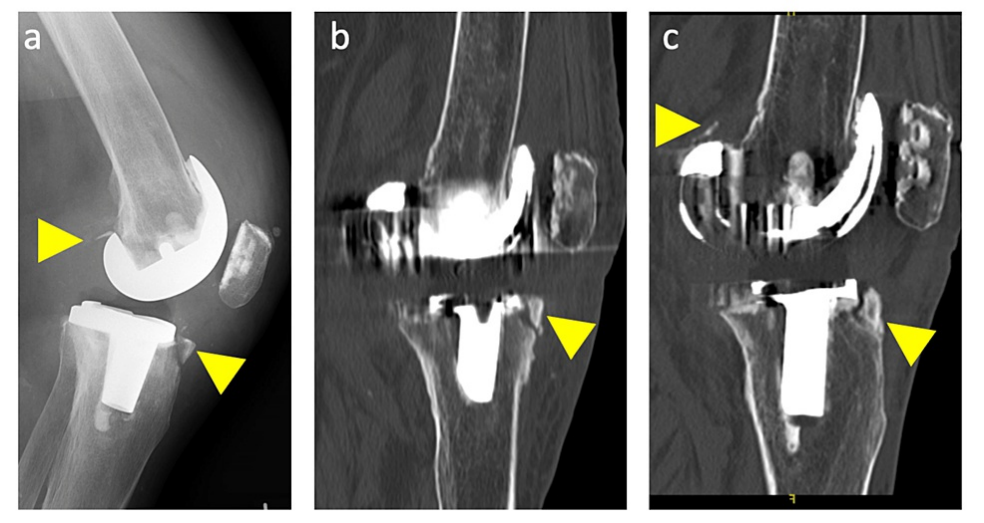
Knee images after fall. Lateral knee radiograph (a) and computed tomography images (b and c) after fall showed avulsion fractures (yellow arrows) at anterior aspect of the tibia and posterior aspect of the femur.

**Figure 3:**
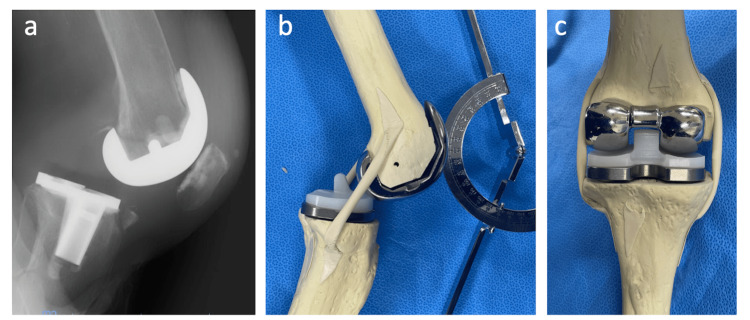
Images of sulubluxation at mid-flexion. Lateral posterior stress radiograph under fluoroscopy at 40° flexion position (a) showed severe instability with posteriorly subluxated tibia. The same size of posterior-stabilized implants simulated in model bone in 40° flexion position showed no post-cam engagement (b, c).

Approximately two months after the left TKA, the patient fell and then became aware of giving way while standing up, walking, and climbing stairs in her left knee. She had anterior knee pain and a swollen knee on the left side. She also had pain during movement. She needed to move carefully not to subluxate her left knee, and she became unable to walk outside. Lateral knee radiographs and CTs showed avulsion fractures of the anterior side of the proximal tibia and the posterior side of the femur (Figure 2a-2c). Varus and valgus stress radiographs showed no apparent ligament failure with either a 150 N varus or valgus load, respectively (Telos GmbH, Laubscher, Holstein, Switzerland), and no significant medial instability was observed in the epicondylar view with a lateral traction force of 50 N applied perpendicular to the lower leg at 80° knee flexion [10]. Subsequently, dynamic imaging was performed using a fluoroscope. No anteroposterior instability was observed in both extension and 90° flexion positions, but her tibia subluxated posteriorly between 40° and 60°, and mid-flexion instability was observed (Figure 3a). No post-cam engagement and instability in the anteroposterior direction were confirmed in these flexion angles using a bone model with the same prosthesis (Figure 3b-3c). Conservative treatment with a functional knee brace and quadriceps training was initiated due to the patient’s hesitation about undergoing a second surgery; however, no improvement was observed. Eventually, revision surgery was planned three months after the fall incident (three months after the left primary TKA). Before revision surgery, the ROM was 0-100° in the left knee, and the KSS-KS and FS of the left knee were 39 and 0 points, respectively.

**Figure 4:**
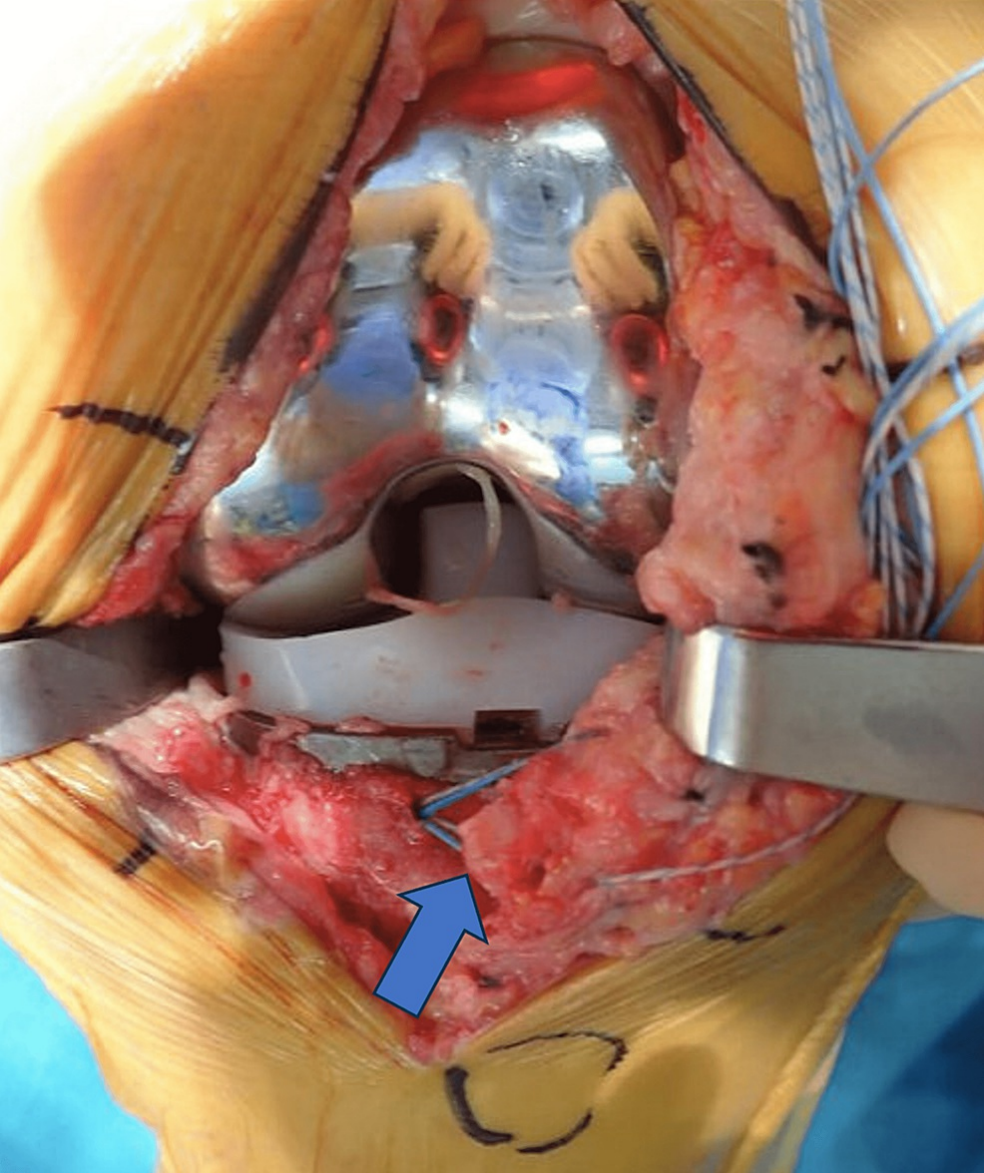
Intraoperative image. Intraoperative image show that the avulsion fragment (blue arrow) was anchored to the tibia, which did not provide sufficient stability.

In the revision surgery, a fractured bone fragment with dimensions of 30 mm × 10 mm × 6 mm in front of the tibial component was observed (Figure 4). The lateral side was hinged, and the fractured bone fragment was unstable. First, fixation of the bone fragment to the tibia was performed with three soft anchors (Juggerknot, Zimmer-Biomet) inserted into the tibial side, and sutures were passed through the articular capsule. The polyethylene insert was removed and exchanged for a thicker trial insert of 17 mm; subsequently, a dynamic evaluation under fluoroscopy was performed with the bone fragment stabilized to the tibia. Anteroposterior instability diminished, and posterior subluxation was prevented; however, mild posterior instability of the tibia remained. We judged that osseous fixation with a thick tibial insert was insufficient to resolve the instability. Fixation of the posterior femoral avulsion fracture might provide more instability; however, it needed an additional posterior approach, and we thought that it would be less promising than revision TKA with a rotating-hinge prosthesis. Thus, revision TKA was performed using the rotating-hinge knee implant to obtain secure anteroposterior stability (Zimmer-Biomet, NexGen Rotating Hinge Knee, tibial insert 14 mm). After the femoral component was removed, avulsion of the posterior capsule was confirmed. Since we decided to use a hinge prosthesis, no additional procedure was performed on the posterior capsule.

**Figure 5:**
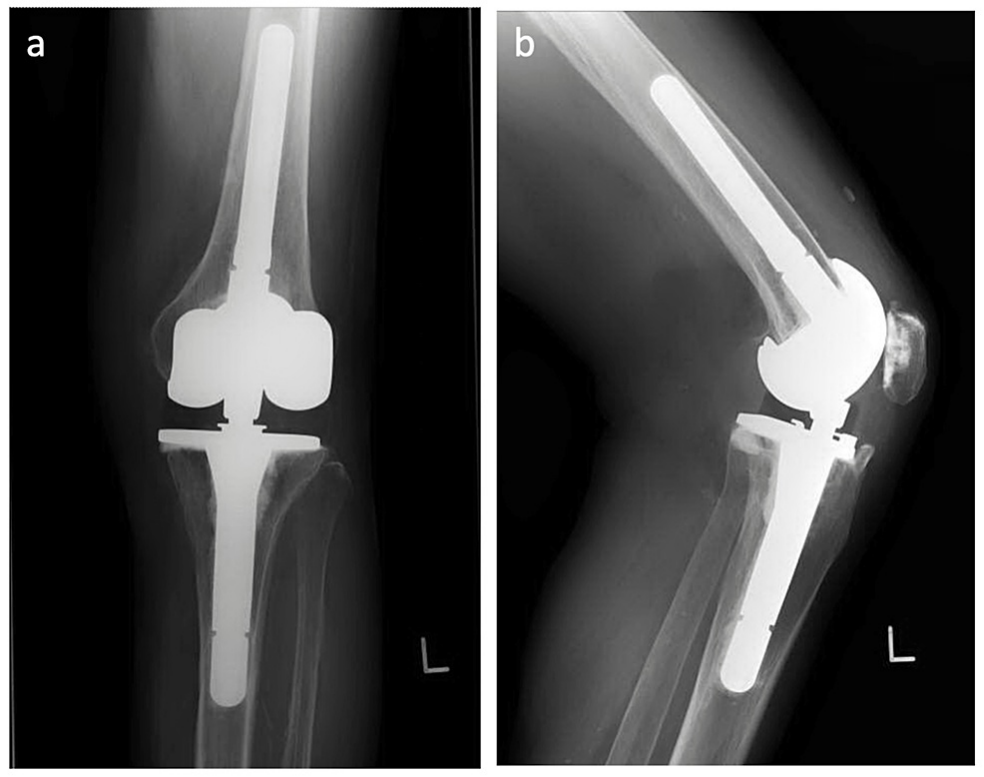
Postoperative radiographs. Anterior-posterior (a) and lateral radiographs (b) at 2 years after revision total knee arthroplasty.

The postoperative course was uneventful, and she was able to walk with a cane within two weeks after revision TKA with no complaints of instability. Two years after revision TKA, the patient could walk with a cane without a recurrence of a sense of instability. The bone union of the tibial avulsion fracture was confirmed, with no implant complications (Figure 5). The ROM was 0-125° and 0-120° in the left and right knees, respectively. KSS-KS improved to 100 and 100 points, and FS improved to 65 and 65 points in the left and right knees, respectively.

## Discussion

We reported a case of severe mid-flexion instability in PS-TKA after traumatic avulsion fractures of the tibia and femur. Stabilization of the tibial avulsion fracture and thickening of the insert did not sufficiently stabilize the instability, and revision TKA with a rotating-hinge prosthesis provided pain relief and functional recovery.

Mid-flexion instability was first reported in 1990 by Martin and Whiteside in a cadaveric study, which indicated that instability occurs in the intermediate flexion position between 0° and 90° but not in the extension or flexion position of 90° or more [11]. In mid-flexion instability, patients often notice instability, especially when they get up from a chair or go up and down a staircase. In a previous report, instability was noticed when instability in the anteroposterior direction was 7 mm or more [6]. In this case, the instability in the anteroposterior direction was far greater than 7 mm between 40° and 60°.

Causes of mid-flexion instability include patient-related factors (traumatic falls postoperatively, a history of connective tissue disorders, or neuromuscular disorders), implant-related factors (implant type and design, wear and bone loss/osteolysis causing loosening of implants, and gap imbalance), and technique-specific factors (malalignment of the implant, malrotation, ligament failure, and failure to balance the knee coronally through either under-release or inadvertent over-release of the soft tissue envelope) [12,13]. Although the PS implant of "J-curve" design, used in this study, is vulnerable to causing mid-flexion instability [14], given that there were no instability-related symptoms before traumatic falls, fracture of the anterior aspect of the tibia and posterior femur plausibly caused the joint capsule to detach from the bone, resulting in anteroposterior instability.

In the PS implant, during deep flexion, the cam of the femoral component contacts the post of the insert, which acts as a substitute for the posterior cruciate ligament, consequently preventing the back movement of the tibia. The angle of post-cam engagement depends on the design of the prosthesis and the angle of implant fixation, but for the implant in this study, it is about 75°. The roll-back function also enables deep bending. In the case of subluxation in the mild flexion position, a posterior force acts on the tibia by contraction of the hamstring, and the cam of the femur component passes through the post of the insert [15]. In addition, we examined the positional relationship between the cam and post in the extension, mid-flexion, and 90° flexion positions using the same implant that we used in the primary TKA. We found that in flexion at more than about 70°, the cam of the femur component and the post of the insert made firm contact, and posterior dislocation did not occur. However, in the flexion of about 40-60°, where patients showed tibial posterior subluxation, the cam and post were not in contact. As the reason for the mid-flexion instability, we speculate that knee stability was enforced by the tension of the joint capsule at these flexion angles, but the stabilization was diminished by the breakdown of the joint capsules due to an avulsion fracture of the attachment, eventually leading to instability. To stabilize the joint capsule and small bony fragment, the fixation with anchors may not have been insufficient. However, the bony fragment was too small for screw fixation. Although the enforcement of an autograft and/or artificial ligament is useful in the treatment of the extensor mechanism, it seemed difficult to effectively enforce the thinner joint capsule with those materials compared with the patellar tendon.

The treatment of instability after TKA includes conservative therapy, such as physical therapy, drug therapy, bracing, and surgical therapy, such as revision TKA. It has been reported that 66% of patients with flexion instability after TKA do not improve with conservative treatment [7]. Revision to a thicker polyethylene liner is one approach, but this narrows the space for both extension and flexion and increases the risk of flexion contracture in the long term [16]. The long-term results of revision TKA using a rotating-hinge implant have been good, and a 10-year survival rate of ≥90% has been reported [17]. As a limitation, the tibial component was placed in a varus position, and HKA was 3.8° varus after the initial surgery, although mechanical alignment was aimed. However, there was no varus/valgus instability before revision surgery, or patients showed good recovery and improved until the traumatic event. Considering these things, we thought mid-flexion instability was mainly caused by trauma, not by the initial surgical error. Nevertheless, the coronal alignment error or the implant design, which is vulnerable to mid-flexion instability, might have some effects on the instability when combined with avulsion fractures.

## Conclusions

In the present case report, catastrophic mid-flexion instability occurred after avulsion fractures of the articular capsule of the femur and tibia in a patient with PS TKA. Orthotics and quadriceps training were first provided, but surgical intervention was required due to modest improvement. Intraoperatively, fixation of the tibial fracture and thickening of the insert were not enough to improve the instability in the mid-flexion range. With the revision of the rotating-hinge prosthesis, stability was obtained, and the patient showed good improvement. Loss of support by the joint capsules due to an avulsion fracture may cause significant anteroposterior instability in the mid-flexion position.
